# Book Reviews

**Published:** 1799-03

**Authors:** 


### POPULAR OR DOMESTIC MEDICINE.

*Du Régime diététique, &c.* "*Of Dietetic Regimen in the cure of Diseases:*" by C. J. TISSOT. *Strasbourg.* Koenig.

This treatise is divided into three principal sections, in each of which are to be found a number of sound and practical remarks on the most efficacious and least complicated methods of preserving health: in the first, the venerable and celebrated author has classed the usual articles of food and drink, according to their comparative salubrity; in the second, he lays down general rules rules of diet, in reſpect to the temperament, ſex, habit, paſſions, and other circumſtances of the patient, the climate, ſeaſon, &c.: and in the third ſection the cauſes of thoſe diſeaſes, to which the dietetic rules chiefly apply, are traced and aſcertained with great learning and judgment.

*Traité ſur la manière d'élever ſainement les enfans,* &c. "*A Treatiſe on the method of rearing healthy children; founded on medical and philoſophical principles.*" By I. P. FRANK, Counſellor of State to the Emperor of Germany, Profeſſor of Chemiſtry at Vienna, &c. *Translated from the German,* by M. BOEHRER, Phyſician at Paris.

This may be conſidered a valuable acceſſion to the various treatiſes lately publiſhed, in England and Germany, on the important ſubject of phyſical education. It is a truly *philoſophic*, benevolent, and ſpirited attempt to overcome and eradicate old-rooted prejudices. Although the author does not always condeſcend to preſent his readers with the moſt eaſy and familiar illuſtrations of the ſubjects in queſtion, we muſt nevertheleſs do him the juſtice to ſay, that in our opinion his treatiſe is not inferior in real merit and utility to the claſſical productions of UNZER, HUFELAND, CAMPE, CLAUDIUS, WURZER, &c.

*Ueber die Erzishung und Behandlung dee Kiuder,* &c. *On the Education and Management of Children, in the firſt years of their Life. A Manual for thoſe Mothers who are concerned in the health of their Offspring, &c.* By Dr. C. A. STRUVE. 8vo. 286 pp. *Hanover.* Hahn.

In this popular treatiſe, the Author ſuggeſts many practical rules for the proper treatment of children, in the ſtate of infancy. He takes a comprehenſive view of all the exerciſes of children tending to promote their health; and as the ſtyle of Dr. Struve is throughout clear, and inſtructive, and his work often entertaining, by the familiar, and ſtriking illuſtrations with which it is interſperſed, readers deſirous of uſeful information, and particularly mothers, will be highly gratified in peruſing this excellent little treatiſe.

To render this performance in every reſpect worthy of the patronage of the guardians of youth, the induſtrious author has not only laid down general precepts for the moſt rational treatment of children, in a phyſical reſpect, but he has likewiſe with great accuracy pointed out the exceptions from theſe general rules, according to their different conſtitution, temperaments, and other circumſtances. Laſtly, the caſes in which the advice of a phyſician, or other medical aſſiſtance becomes neceſſary, are fairly ſtated; and in a ſecond appendix, the amuſements moſt conducive to the health of children, are critically examined and aſcertained.

